# 植物中27种典型药品及个人护理品多残留检测方法的建立及其在芽苗菜中迁移规律的分析

**DOI:** 10.3724/SP.J.1123.2022.09017

**Published:** 2023-05-08

**Authors:** Yongfu ZENG, Meifang CHEN, Yu SHAO, Yonghuan YAN, Haichao ZHANG, Jing WANG, Lianfeng AI, Weijun KANG

**Affiliations:** 1.河北医科大学公共卫生学院,河北石家庄050017; 1. School of Public Health, Hebei Medical University, Shijiazhuang 050017, China; 2.河北省环境与人群健康重点实验室, 河北石家庄050017; 2. Hebei Key Laboratory of Environment and Population Health, Shijiazhuang 050017, China; 3.中国合格评定国家认可委员会,北京100062; 3. China National Accreditation Service for Conformity Assessment, Beijing 100062, China; 4.河北医科大学法医学院,河北石家庄050017; 4. School of Forensic Medicine, Hebei Medical University, Shijiazhuang 050017, China; 5.石家庄海关技术中心,河北石家庄050051; 5. Shijiazhuang Customs Technology Center, Shijiazhuang 050051, China

**Keywords:** 药品及个人护理品, 超高效液相色谱-串联质谱法, 芽苗菜, 迁移因子, pharmaceuticals and personal care products (PPCPs), ultra-performance liquid chromatography-tandem mass spectrometry (UPLC-MS/MS), sprouts, transfer factors

## Abstract

建立了一种基于超高效液相色谱-串联质谱同时检测植物体内27种典型药品及个人护理品(PPCPs)残留的分析方法,经HLB萃取小柱对植物体内PPCPs富集净化,以BEH C18色谱柱(100 mm×2.1 mm, 1.7 μm)分离,以0.1%甲酸水溶液-乙腈为流动相梯度洗脱,电喷雾电离质谱正离子多反应监测(MRM)模式下分析。方法学考察表明,植物体中27种PPCPs的检出限为0.01~0.30 μg/kg;定量限为0.03~0.98 μg/kg;在各自的检测范围内有良好的线性关系(*r*^2^>0.99),平均回收率为80.8%~122.3%, 相对标准偏差 (RSD)为1.0%~9.9%。使用本方法检测生长在不同含量PPCPs培养基中的芽苗菜,结果发现在低含量PPCPs培养基中生长的芽苗菜体内检出了10种PPCPs;在中含量PPCPs培养基中生长的芽苗菜体内检出13种PPCPs;在高含量PPCPs培养基中生长的芽苗菜体内检出19种PPCPs。结果表明在有PPCPs污染的水体中生长的植物或是用含有被PPCPs污染的水源灌溉的植物会吸收并积累PPCPs,且植物对PPCPs的吸收种类及吸收量与外界PPCPs的水平有着密切关系。通过对植物根、茎、叶中PPCPs含量的分析发现,除豪莫西布曲明(叶>茎>根)、格列苯脲(根>叶>茎)外,其余吸收的PPCPs在植物组织间的含量分布均为根>茎>叶,表明PPCPs在植物体内的分布存在差异。通过计算PPCPs在植物体内的迁移因子(TF)发现,不同PPCPs在植物体内的迁移能力有着明显差异,豪莫西布曲明的TF=2.34,氯代西布曲明的TF=1.25,均>1,表明在植物体内发生迁移的药物中,豪莫西布曲明与氯代西布曲明在芽苗菜体内迁移能力最强,盐酸尼卡地平以及马来酸氯苯那敏迁移能力次之,金刚烷胺、*N*-单去甲基西布曲明、卡马西平以及氟甲喹等药物迁移能力最弱。PPCPs一旦被吸收后会转移到植物的茎或(和)叶中积累,并对植物其他器官造成污染而带来潜在危害;因此,在后续研究中应重点关注豪莫西布曲明、氯代西布曲明等PPCPs在植物体内的迁移。

## Abstract (en)

An analytical method based on ultra-performance liquid chromatography-tandem mass spectrometry was developed for the simultaneous determination of 27 pharmaceutical and personal-care product (PPCP) residues in plants. The enrichment and cleanup of PPCPs in plants were achieved using an HLB extraction column, and the separation was performed on a BEH C18 column (100 mm×2.1 mm, 1.7 μm) with 0.1% formic acid water-acetonitrile as the mobile phase via gradient elution. PPCPs were detected with electrospray ionization mass spectrometry in positive-ion multiple-reaction monitoring (MRM) mode. The limits of detection and quantification of the 27 PPCPs in plants were 0.01-0.30 μg/kg and 0.03-0.98 μg/kg, respectively. Good linearities were observed with coefficients of determination (*r*^2^) >0.99. The spiked recoveries were between 80.8% and 122.3% with relative standard deviations (RSDs) between 1.0% and 9.9%. The method was subsequently used to study sprouts grown in different concentrations of PPCPs. A total of 10 PPCPs were detected in sprouts grown in medium with a low concentration PPCPs, 13 PPCPs were detected in sprouts grown in medium with a moderate concentration of PPCPs, and 19 PPCPs were detected in sprouts grown in medium with a high concentration of PPCPs. These results showed that plants grown in water bodies contaminated with PPCPs or irrigated with water contaminated with PPCPs absorbed and accumulated these substances and that the amount and type of PPCPs absorbed by plants were closely related to the levels of PPCPs in the external environment. Analysis of the contents of PPCPs in different plant tissues revealed a general distribution of root>stem>leaf. Haemosibutramine showed a tissue distribution of leaf>stem>root, while glibenclamide showed a distribution of root>leaf>stem; these results revealed differences in the distribution of PPCPs in plants. Calculation of the transfer factor (TF) of the PPCPs in plants demonstrated significant differences in the transferability of different PPCPs, with TF=2.34 for haemosibutramine and TF=1.25 for chlorosibutramine. The results showed that among the drugs that migrated in plants, haemonosibutramine and chlorosibutramine had the strongest migration ability in sprouts, followed by nicardipine and chlorpheniramine maleate, and amantadine, *N*-monodesmethyl sibutramine, carbamazepine and flumequine had the weakest migration ability. Once absorbed, these compounds were transferred to the stems and/or leaves, where they accumulate and cause potential harm by contaminating other plant organs. Therefore, PPCPs such as homosibutramine and chlorosibutramine, which easily migrate in plants, should be given extra attention in future studies. The method is simple in pre-treatment, sensitive and accurate, and can be widely applied to the detection of PPCP residues in plant samples.

药品及个人护理品(pharmaceuticals and personal care products, PPCPs)与人类的生产、生活息息相关,其包括化妆品、洗护用品、消毒剂、降糖药、抗生素、消炎药、降压药及保健品等^[1]^。随着社会的发展,PPCPs的种类及使用量呈逐年剧增的趋势;由于PPCPs在环境中分布广泛,具有迟缓的生物降解性和持久性,被认定为对环境具有潜在威胁的化学物质。据文献[2⇓-4]报道,全球许多国家和地区的环境介质中检出了PPCPs。许多人类及动物常用治疗性药物未经生物体代谢或未完全代谢直接通过粪便、尿液等排泄物进入污水系统,甚至有的未经使用即通过各种途径进入环境之中;个别药物生产厂商还会将非法药物与合成副产物直接排入城市排污系统,例如抗炎药物(对乙酰氨基酚、普奈生、阿司匹林等)^[5,6]^、阻断剂药物(阿替洛尔、苯海拉明、马莱酸氯苯那敏等)^[7]^、血脂调节剂(吉非罗齐、阿托伐他汀钙、美伐他汀、洛伐他汀等)^[7]^、抗癫痫类药物(卡马西平、地兰汀等)^[8]^、兴奋剂类药物(咖啡因、可的松及丙酸睾酮等)、血压调节剂(尼群地平、尼莫地平等)、降血糖药物(格列美脲、格列齐特等)、抗生素(三氯卡班、氟甲喹、司帕沙星等)^[9]^以及减肥类药物(西布曲明、*N*-单去甲基西布曲明等)均在人类活动的水体环境中常有检出,且检出水平从1 μg/L至140 μg/L不等^[10]^。

在一些研究中,用处理后的废水灌溉植物,PPCPs可以经过植物根进入植物体内^[11,12]^。例如,在一项用处理后废水灌溉苹果树的研究^[7]^中发现在叶片中检出了5种PPCPs,含量为13.9~532 ng/g(鲜重)。向环境水体中持续输入PPCPs,不仅会对环境水体及生物带来潜在的危害,而且还会对饮水安全以及食品安全造成影响^[13]^。

目前,已有较多文献报道了利用高效液相色谱-串联质谱仪(HPLC-MS/MS)对环境介质(土壤、污泥以及污水等)^[14⇓⇓⇓⇓-19]^中的抗生素、抗炎药、阻断剂药物等新型污染物进行微量分析。但植物样本与水体、土壤样本相比,组分更为复杂,植物中往往含有大量的天然色素、脂质以及蛋白质,会导致严重的基质干扰,影响分析的灵敏度^[20]^。对于植物样本中多种类PPCPs的分析,一种有效的样本制备处理方法结合最佳色谱、质谱条件可以在很大程度上降低基质干扰,同时能够确保PPCPs获得最大的回收率,保证检测结果的准确性。

本研究以27种人类疾病常用治疗性药物作为PPCPs的研究分析对象,其中包括降血压药物、降血脂药物、减肥药、抗生素及抗病毒药物。结合超声提取,SPE富集净化及LC-MS/MS建立了高灵敏度的植物体内药物残留检测分析方法。同时在恒温恒湿植物培养箱中,以不同含量水平的PPCPs培养液、水培育芽苗菜,并考察了水培芽苗菜对PPCPs的吸收、迁移以及积累。

## 1 实验部分

### 1.1 仪器、试剂与材料

LCMS-8050液相色谱-质谱联用仪(日本SHIMADZU公司); Milli-Q超纯水制备仪(美国Millipore公司); HYM-1500-S恒温恒湿箱(上海沪粤明科学仪器有限公司)。Oasis HLB固相萃取柱(150 mg/6 mL, Waters公司)。

27种PPCPs标准物质,包含尼索地平(Nis,纯度99.0%)、尼群地平(Nit,纯度99.4%)、尼莫地平(Nim,纯度99.6%)、盐酸尼卡地平(Nic,纯度98.0%)、米诺地尔(Min,纯度99.7%)、吲达帕胺(Ind,纯度99.1%)、洛伐他汀(Lov,纯度99.7%)、美伐他汀(Mev,纯度98.6%)、金刚烷胺(Ama,纯度98.5%)、苯海拉明(Dip,纯度99.9%)、马来酸氯苯那敏(Chl,纯度99.8%)、卡马西平(Car,纯度99.5%)、西布曲明(Sib,纯度99.5%)、氯代西布曲明(Chl-sib,纯度97.2%)、豪莫西布曲明(Hom-sib,纯度95.4%)、*N*-单去甲基西布曲明(*N*-Sib,纯度99.7%)、*N*,*N*-双去甲基西布曲明(*N*,*N*-Sib,纯度99.2%)、格列苯脲(Glibe,纯度99.5%)、格列美脲(Glim,纯度99.6%)、格列齐特(Glic,纯度99.5%)、瑞格列奈(Rep,纯度99.8%)、达那唑(Dan,纯度99.2%)、丙酸睾酮(Oreton,纯度99.9%)、可的松(Cor,纯度98.9%)、阿托伐他汀钙(Ato,纯度95.0%)、氟甲喹(Flu,纯度99.3%)以及司帕沙星(Spa,纯度99.4%)均购于北京曼哈格生物科技有限公司,结构式见图1。

**图1:**
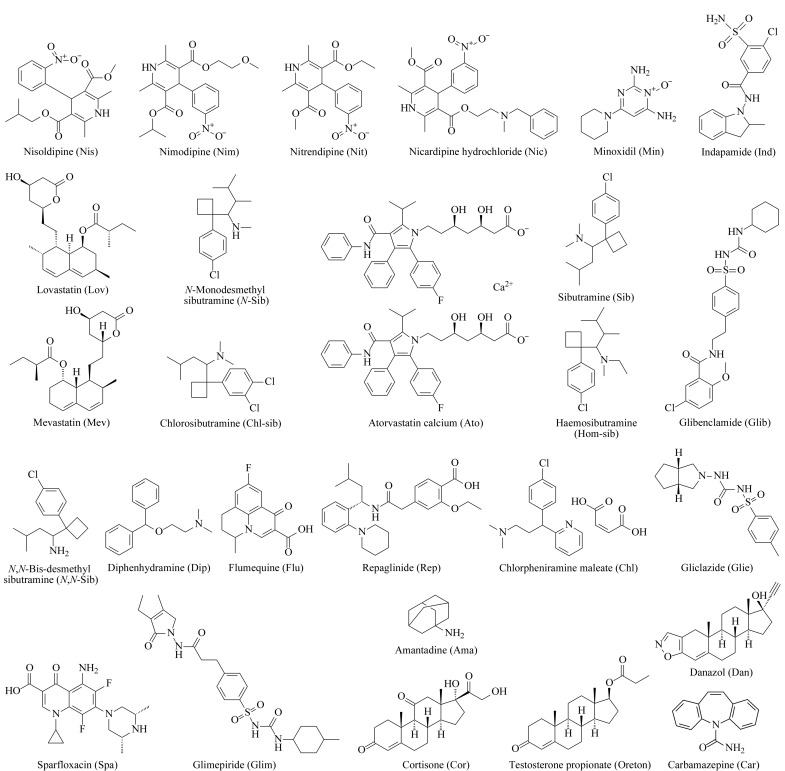
27种PPCPs的结构式

乙腈为色谱纯,购自美国Fisher公司;甲醇为色谱纯,购自上海Anpel公司;甲酸为色谱纯,购自美国DIKMA公司;氯化钠为分析纯,购自天津市永大化学试剂有限公司;去离子水由Milli-Q超纯水制备仪制备。

### 1.2 标准溶液配制

标准储备液的配制:准确称取尼索地平、尼莫地平、尼群地平、盐酸尼卡地平、米诺地尔、吲达帕胺、洛伐他汀、美伐他汀以及金刚烷胺各0.01 g于10 mL容量瓶中,用甲醇配制成质量浓度为1 g/L的混合标准储备液;准确称取苯海拉明、马来酸氯苯那敏、卡马西平、西布曲明、氯代西布曲明、*N*-单去甲基西布曲明、*N*,*N*-双去甲基西布曲明、豪莫西布曲明以及阿托伐他汀钙各0.01 g于10 mL容量瓶中,用甲醇配制成质量浓度为1 g/L的混合标准储备液;准确称取格列苯脲、格列美脲、格列齐特、瑞格列奈、可的松、达那唑、丙酸睾酮、氟甲喹以及司帕沙星各0.01 g于10 mL容量瓶中,用甲醇配制成质量浓度为1 g/L的混合标准储备液。储备液置于-20 ℃保存,备用。

标准工作液的配制:将混合标准储备液用适量甲醇稀释成所需浓度,临用新制。

### 1.3 样品前处理

将芽苗菜样本匀浆,取2 g于50 mL离心管中,然后加入5 g氯化钠,20 mL乙腈,涡旋混匀,再超声提取20 min, 10000 r/min离心5 min,取上层乙腈溶液5 mL,使用氮气吹至近干,加入1 mL甲醇复溶,再加入9 mL超纯水混匀。然后通过用7 mL甲醇和7 mL去离子水预处理的HLB固相萃取柱中,用5 mL超纯水淋洗小柱,再用7 mL甲醇洗脱提取物;将甲醇提取物置于40 ℃水浴中,使用氮气吹至近干,残余物用1 mL 0.1%甲酸水溶液-乙腈(1∶1, v/v)复溶,过0.22 μm尼龙滤膜,上机待测。

### 1.4 仪器条件

液相色谱条件:Waters ACQUITY UPLC BEH C18色谱柱(100 mm×2.1 mm, 1.7 μm),流动相A为0.1%甲酸水溶液,流动相B为乙腈,流速为0.4 mL/min。梯度洗脱程序:0~0.5 min, 10.0%B; 0.5~6 min, 10.0%B~90.0%B; 6~8 min, 90.0%B; 8~8.1 min, 90.0%B~10.0%B; 8.1~10.1 min, 10.0%B。柱温35 ℃,自动进样器温度保持在4 ℃,进样体积为5 μL。

质谱条件:电喷雾电离源(ESI);正离子扫描模式;多反应监测(MRM);离子源温度:300 ℃;碰撞气:氩气;干燥气:氮气;雾化气流速(nebulizing gas flow): 3.0 L/min。27种PPCPs化合物及质谱检测参数见表1。

**表1:** 27种PPCPs化合物及其质谱检测参数

Drug	CAS number	Drug type	Ionization mode	Parent ion (*m/z*)	Daughter ion (*m/z*)	Collision energy/eV	*t*_R_/ min
Nisoldipine (尼索地平)	63675-72-9	antihypertensive drug	[M+H]^+^	389.2	239.2^*^/195.2	21/42	6.17
Nimodipine (尼莫地平)	66085-59-4	antihypertensive drug	[M+H]^+^	419.2	343.2^*^/301.1	14/26	3.12
Nitrendipine (尼群地平)	39562-70-4	antihypertensive drug	[M+H]^+^	361.2	315.1^*^/329.2	14/15	5.85
Nicardipine hydrochloride	54527-84-3	antihypertensive drug	[M+H]^+^	480.3	315.2^*^/359.2	25/25	4.51
(盐酸尼卡地平)							
Minoxidil (米诺地尔)	38304-91-5	antihypertensive drug	[M+H]^+^	210.2	164.2^*^/110.1	22/25	3.14
Indapamide (吲达帕胺)	26807-65-8	antihypertensive drug	[M+H]^+^	366.1	132.1^*^/91.1	14/44	4.72
Lovastatin (洛伐他汀)	75330-75-5	lipid-lowering drug	[M+H]^+^	459.3	357.3^*^/342.2	24/26	5.67
Mevastatin (美伐他汀)	73573-88-3	lipid-lowering drug	[M+H]^+^	391.3	185.2^*^/159.1	12/28	6.48
Atorvastatin calcium (阿托伐他汀钙)	134523-03-8	lipid-lowering drug	[M+H]^+^	559.3	440.3^*^/292.3	23/33	5.71
Sibutramine (西布曲明)	106650-56-0	weight-reducing aid	[M+H]^+^	280.2	125.1^*^/139.1	25/15	4.75
Chlorosibutramine (氯代西布曲明)	84485-08-5	weight-reducing aid	[M+H]^+^	314.2	159.0^*^/173.0	27/17	4.92
*N*-Monodesmethyl sibutramine	84467-94-7	weight-reducing aid	[M+H]^+^	266.2	125.1^*^/139.1	24/14	4.65
(*N*-单去甲基西布曲明)							
*N*,*N*-Bis-desmethyl sibutramine	84484-78-6	weight-reducing aid	[M+H]^+^	252.2	125.0^*^/139.1	22/12	4.58
(*N*,*N*-双去甲基西布曲明)							
Haemosibutramine (豪莫西布曲明)	935888-80-5	weight-reducing aid	[M+H]^+^	294.3	125.1^*^/139.1	26/17	4.85
Glibenclamide (格列苯脲)	10238-21-8	hypolycemic agent	[M+H]^+^	494.2	369.1^*^/169.1	15/36	5.78
Glimepiride (格列美脲)	93479-97-1	hypolycemic agent	[M+H]^+^	491.3	352.2^*^/126.1	15/27	5.94
Repaglinide (瑞格列奈)	135062-02-1	hypolycemic agent	[M+H]^+^	453.3	230.2^*^/162.2	28/21	4.95
Gliclazide (格列齐特)	21187-98-4	hypolycemic agent	[M+H]^+^	324.2	127.2^*^/110.1	18/22	5.43
Diphenhydramine (苯海拉明)	58-73-1	antihistamine drug	[M+H]^+^	256.2	167.2^*^/152.1	14/37	4.19
Chlorpheniramine maleate	113-92-8	antihistamine drug	[M+H]^+^	275.2	230.1^*^/167.1	17/38	3.61
(马来酸氯苯那敏)							
Flumequine (氟甲喹)	42835-25-6	antibacterial drug	[M+H]^+^	262.1	244.1^*^/220.0	21/8	4.72
Sparfloxacin (司帕沙星)	110871-86-8	antibacterial drug	[M+H]^+^	393.2	292.1^*^/149.0	26/55	3.45
Amantadine (金刚烷胺)	768-94-5	antiviral drug	[M+H]^+^	180.2	163.2^*^/81.1	17/23	3.73
Carbamazepine (卡马西平)	298-46-4	antiepileptic drug	[M+H]^+^	237.2	194.2^*^/179.2	19/34	4.56
Cortisone (可的松)	53-06-5	adrenocortical hormone	[M+H]^+^	361.2	163.1^*^/121.1	23/29	4.24
Testosterone propionate (丙酸睾酮)	57-85-2	androgens	[M+H]^+^	345.2	97.1^*^/109.1	24/31	7.24
Danazol (达那唑)	17230-88-5	antiestrogenic drug	[M+H]^+^	338.3	120.1^*^/148.2	30/25	6.29

* Quantitative ion.

## 2 结果与讨论

### 2.1 色谱条件的优化

#### 2.1.1 色谱柱

考察了SHISIDO ADME色谱柱(150 mm×2.1 mm, 2.7 μm)、Waters ACQUITY UPLC BEH C18色谱柱(100 mm×2.1 mm, 1.7 μm)、ACQUITY UPLC HSS T3色谱柱(100 mm×2.1 mm, 1.8 μm)以及Thermo Hypersil GOLD色谱柱(150 mm×2.1 mm, 5 μm) 4种色谱柱对27种PPCPs分离效果的影响。发现阿托伐他汀钙、西布曲明、达那唑、卡马西平、金刚烷胺等化合物在SHISIDO ADME色谱柱以及Thermo色谱柱下的峰形不佳,峰较宽,出现较严重的拖尾峰;而在ACQUITY UPLC HSS T3色谱柱上各物质峰形良好,但尼索地平、尼莫地平、金刚烷胺等化合物的响应比在Waters ACQUITY UPLC BEH C18色谱柱上的响应低。因此,选用Waters ACQUITY UPLC BEH C18色谱柱用于PPCPs分析,此时峰形最佳,且响应良好。

#### 2.1.2 流动相

考察了水-乙腈、水-甲醇、0.05%甲酸水溶液-乙腈、0.1%甲酸水溶液-乙腈以及0.2%甲酸水溶液-乙腈作为色谱流动相的分离效果。结果表明,与甲醇相比,乙腈作为流动相,大部分药物有较好的响应,大多数化合物的敏感性提高。与水相中不添加甲酸相比,加入甲酸后大部分物质的响应有一定的提升,且峰形得到改善;进一步对水相中甲酸的体积分数进行优化,发现采用0.1%甲酸水溶液,大部分物质的响应最佳。因此,选用0.1%甲酸水溶液-乙腈作为流动相,此时能够得到很好的分离效果。

### 2.2 质谱条件的优化

将0.2 mg/L PPCPs混合标准溶液注入质谱仪中,以确定目标化合物的最佳毛细管温度、离子源温度、特征子离子、Q1电压、Q3电压、氮气流速、碰撞能量等质谱条件。根据目标化合物的相对分子质量,设定合适的质谱扫描范围,对母离子进行正、负模式下的全扫描质谱分析,其中各物质的分子离子峰[M+H]^+^响应值最高。在正离子模式下进行子离子扫描,从而得到主要的子离子碎片,并对响应值高的子离子碎片进行碰撞能优化,确定最终的反应监测离子对、碰撞能以及相应参数(见表1)。

### 2.3 提取溶剂的选择

选择合适的提取溶剂可以去除一部分干扰杂质,提高检测准确度。根据文献[21,22]报道,植物样本中药物的常用提取溶剂主要有乙腈、甲醇、乙酸乙酯及其酸化溶液。本实验以空白芽苗菜为实验对象,在50 μg/kg药物添加水平下,重复6次,对比考察乙腈、甲醇、乙酸乙酯、0.1%甲酸乙腈作为提取溶剂的提取效果。结果发现,甲醇、乙酸乙酯作为提取溶剂时,一部分药物的平均回收率低于50.0%,提取效果较差;而乙腈和0.1%甲酸乙腈作为PPCPs的提取溶剂时,PPCPs的回收率为69.9%~106.5%,两种提取溶剂对各药物的提取效果差异不大(见图2)。综合考虑成本及操作难易程度,选择乙腈作为提取溶剂,此时PPCPs有良好的提取效果,能够满足实验需求。

**图2:**
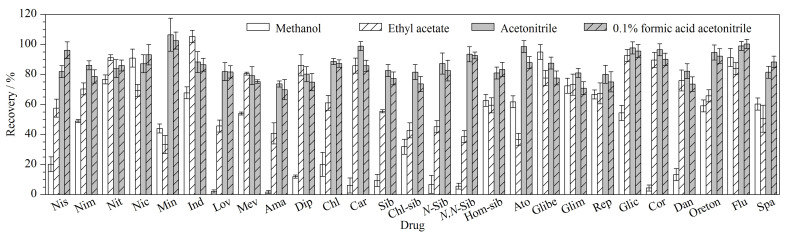
27种PPCPs在不同提取溶剂中的提取效果(*n*=6)

适量的提取溶剂可以大大提高药物的提取效率,同时可以减少有机废液的产生。向空白芽苗菜中准确加入一定体积的标准混合溶液,充分混匀,静置20 min;再分别加入10、15、20、25、30 mL乙腈溶液,充分振荡提取后,收集上层乙腈溶液,经分析测定后,评价不同体积乙腈中药物的平均回收率变化情况(见图3)。当乙腈体积达到20 mL时,PPCPs的提取效率均大于78.9%,当提取溶剂达到25 mL和30 mL时,各药物的回收率变化均在5%以内。因此,从节约环保的角度考虑,选择20 mL乙腈作为提取溶剂,此时各药物均有良好的回收率。

**图3:**
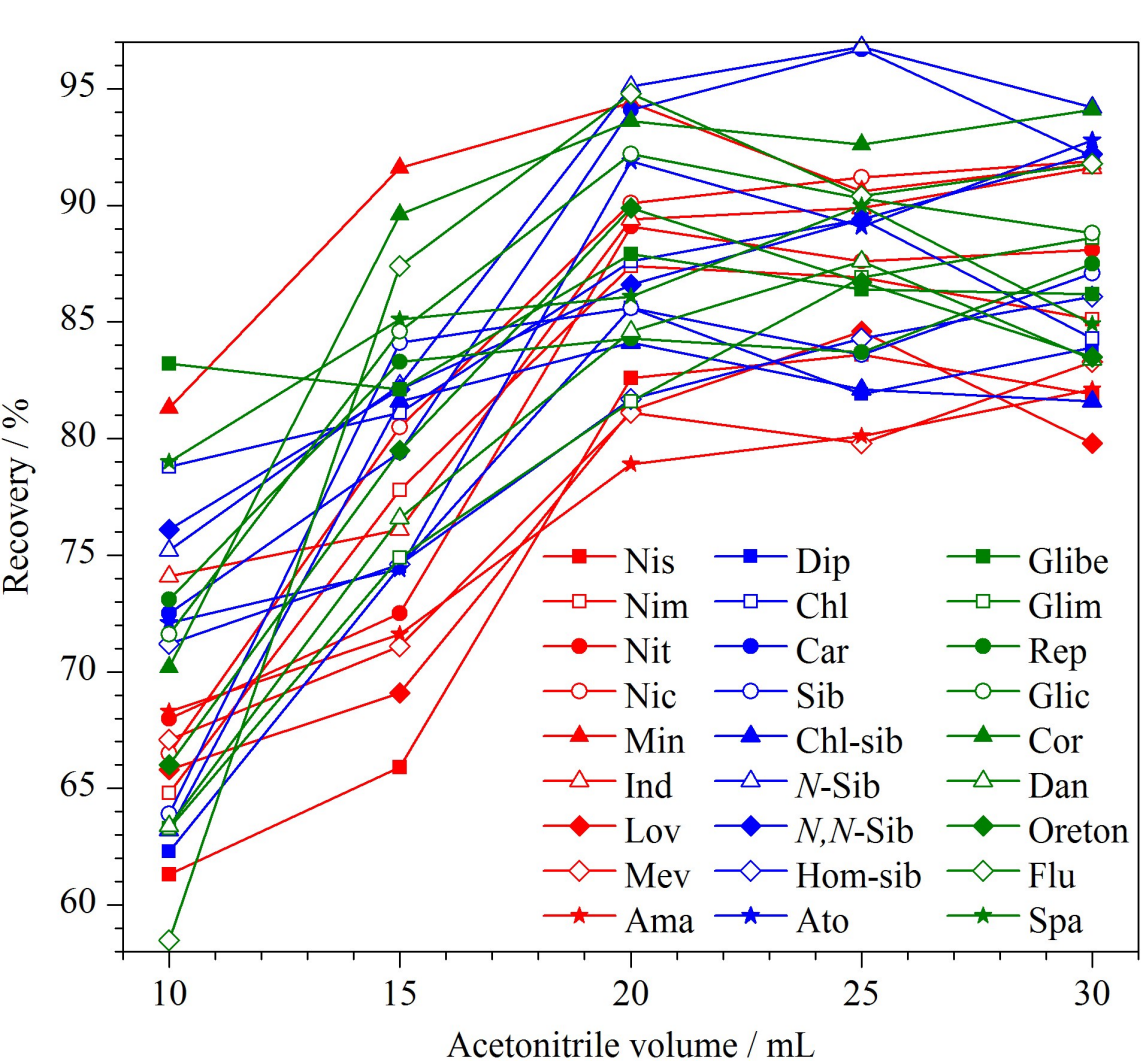
27种PPCPs采用不同体积乙腈提取时的回收率

### 2.4 净化条件的选择

植物样本中含有大量的叶绿素、磷脂以及糖类等物质,会对目标物分析以及色谱柱使用寿命造成不同程度影响,因此,需对提取液进行净化。选择合适的固相萃取小柱可以大大提高净化效率,去除部分干扰杂质,同时还可以减少净化过程中的损失。目前,文献中报道的植物样本净化所选用的固相萃取小柱主要包括混合型阴离子交换固相萃取柱(MAX)、混合型阳离子交换固相萃取柱(MCX)、亲水亲脂平衡固相萃取柱(HLB)等^[23,24]^。本实验通过在空白样品中添加一定浓度的PPCPs标准溶液,考察MAX、MCX、HLB固相萃取小柱在各自最佳使用条件下的萃取效果。实验发现使用HLB固相萃取小柱时,PPCPs的平均回收率大于71.8%,而使用MAX、MCX固相萃取小柱时部分PPCPs的平均回收率低于60.0%。因此,本实验选用HLB固相萃取小柱作为PPCPs的净化小柱,能够满足本实验的要求(见图4)。

**图4:**
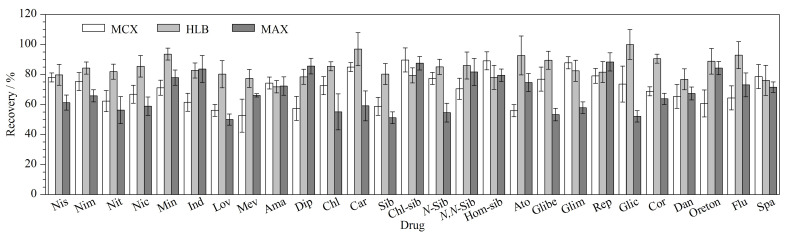
不同净化小柱的净化效果(*n*=6)

### 2.5 基质效应

基质效应(ME)是指样品中除目标化合物以外的其他成分对目标化合物分析造成干扰的现象,一般有基质增强和基质抑制两种情况。本实验以芽苗菜空白基质匹配标准溶液5、50、150 μg/L 3个水平与所对应质量浓度的纯溶剂标准溶液进行比较。ME计算公式:ME=(*B/A*)×100%(*A*为纯溶剂标准溶液的响应值;*B*为空白基质匹配标准溶液的响应值);若80%≤ME≤120%,基质效应可以忽略;若ME>120%或ME<80%,则认为存在基质增强效应或基质抑制效应^[25]^。结果表明,盐酸尼卡地平、金刚烷胺、苯海拉明以及可的松的基质效应>120%,存在基质增强效应;司帕沙星基质效应<80%,存在基质抑制效应。其余化合物基质效应为80%~120%,基质效应不明显(见表2)。消除基质效应的方法主要为基质匹配标准溶液法和同位素内标法,考虑到各化合物之间性质各异,同位素内标无法完全对应,且价格高昂。因此,选择基质匹配外标曲线对样品中的分析物进行定量分析,可以消除基质效应的干扰。

**表2:** 27种PPCPs的基质效应(*n*=3)

Drug	Spiked levels/ (μg/L)	MEs/%	RSDs/%	Drug	Spiked levels/ (μg/L)	MEs/%	RSDs/%
Nisoldipine	5/50/150	116.1/109.3/96.4	6.1/7.8/5.5	*N*-Monodesmethyl	5/50/150	115.1/119.0/94.5	5.4/5.2/5.9
Nimodipine	5/50/150	97.3/85.7/81.2	6.9/5.3/4.8	sibutramine			
Nitrendipine	5/50/150	116.1/101.2/96.1	4.6/7.7/4.4	*N*,*N*-Bis-desmethyl	5/50/150	106.2/98.1/95.7	5.3/2.8/7.2
Nicardipine	5/50/150	156.8/149.0/137.9	5.1/7.4/2.1	sibutramine			
hydrochloride				Haemosibutramine	5/50/150	119.4/112.8/94.7	5.6/2.9/2.6
Minoxidil	5/50/150	91.3/85.4/87.1	4.2/2.7/3.4	Atorvastatin calcium	5/50/150	64.8/69.3/67.8	4.4/6.3/8.3
Indapamide	5/50/150	106.7/92.7/94.1	4.8/6.5/2.6	Glibenclamide	5/50/150	86.5/96.3/97.7	3.7/5.7/4.6
Lovastatin	5/50/150	119.2/118.2/114.9	6.1/5.8/3.3	Glimepiride	5/50/150	93.2/108.7/102.8	2.9/6.1/8.7
Mevastatin	5/50/150	90.2/88.9/84.7	3.8/3.7/2.5	Repaglinide	5/50/150	104.7/104.6/98.0	4.5/3.5/4.8
Amantadine	5/50/150	130.0/147.3/136.5	3.7/4.2/8.1	Gliclazide	5/50/150	103.9/97.3/94.2	4.5/3.4/2.7
Diphenhydramine	5/50/150	138.5/130.5/127.5	6.1/3.9/5.7	Cortisone	5/50/150	124.8/123.9/127.6	6.9/5.1/2.2
Chlorpheniramine	5/50/150	107.0/104.3/96.5	4.9/5.1/6.2	Danazol	5/50/150	95.1/98.4/82.1	6.6/4.3/2.8
maleate				Testosterone	5/50/150	102.3/96.6/90.8	7.4/5.4/8.8
Carbamazepine	5/50/150	95.1/97.2/82.5	2.7/6.2/3.3	propionate			
Sibutramine	5/50/150	90.3/92.7/94.7	3.1/4.1/5.6	Flumequine	5/50/150	105.6/106.9/104.0	3.0/2.1/2.7
Chlorosibutramine	5/50/150	100.7/86.8/87.5	7.9/8.1/6.9	Sparfloxacin	5/50/150	68.2/66.7/63.5	8.2/7.1/4.4

### 2.6 线性范围、检出限及定量限

制备系列基质匹配混合标准溶液,以定量离子峰面积为纵坐标(*y*),以对应质量浓度为横坐标(*x*),建立标准曲线(见表3)。在空白基质中采取低水平添加,以*S/N*>3确定方法检出限(LOD),以*S/N*>10确定方法定量限(LOQ)。结果表明,在各化合物对应的线性范围内,均有良好的线性关系,决定系数(*r*^2^)为0.9912~0.9999; PPCPs的检出限为0.01~0.30 μg/kg,定量限为0.03~0.98 μg/kg。

**表3:** 27种PPCPs的线性方程、线性范围、决定系数、检出限和定量限

Drug	Linear equation	Linear range/(μg/L)	*r*^2^	LOD/(μg/kg)	LOQ/(μg/kg)
Nisoldipine	*y*=1.1×10^5^*x*-1.5×10^4^	0.5-200	0.9997	0.18	0.55
Minoxidil	*y*=4.7×10^5^*x*+2.2×10^5^	0.1-150	0.9997	0.10	0.28
Nimodipine	*y*=3.7×10^4^*x*+4.8×10^4^	1.0-200	0.9999	0.24	0.72
Nicardipine hydrochloride	*y*=1.1×10^5^*x*+1.9×10^5^	0.1-200	0.9940	0.18	0.55
Indapamide	*y*=7.2×10^4^*x*-1.6×10^4^	0.5-200	0.9978	0.20	0.62
Lovastatin	*y*=1.4×10^4^*x*+3.3×10^3^	1.0-100	0.9970	0.28	0.84
Mevastatin	*y*=2.7×10^4^*x*-1.4×10^4^	0.5-100	0.9980	0.25	0.74
Amantadine	*y*=1.2×10^5^*x*+4.2×10^5^	0.1-200	0.9983	0.18	0.55
Diphenhydramine	*y*=2.1×10^5^*x*-1.2×10^5^	1.0-300	0.9960	0.15	0.46
Chlorpheniramine maleate	*y*=5.2×10^5^*x*+4.7×10^5^	0.1-200	0.9971	0.09	0.28
Carbamazepine	*y*=5.6×10^6^*x*+7.5×10^5^	0.1-100	0.9963	0.01	0.03
Sibutramine	*y*=2.9×10^4^*x*+1.7×10^3^	1.0-200	0.9980	0.25	0.74
Haemosibutramine	*y*=7.8×10^5^*x*+4.6×10^5^	0.5-150	0.9984	0.06	0.18
Chlorosibutramine	*y*=1.7×10^5^*x*-5.0×10^5^	1.0-100	0.9962	0.18	0.56
*N*-Monodesmethyl sibutramine	*y*=8.8×10^3^*x*+7.4×10^3^	0.5-100	0.9981	0.30	0.98
*N*,*N*-Bis-desmethyl sibutramine	*y*=4.2×10^5^*x*+1.1×10^5^	0.5-100	0.9997	0.10	0.30
Glibenclamide	*y*=6.8×10^4^*x*+3.6×10^3^	1.0-150	0.9963	0.20	0.62
Glimepiride	*y*=3.0×10^4^*x*+2.0×10^3^	1.0-150	0.9912	0.25	0.73
Gliclazide	*y*=2.9×10^5^*x*+4.5×10^3^	1.0-100	0.9999	0.15	0.46
Repaglinide	*y*=5.3×10^5^*x*+8.7×10^3^	0.1-100	0.9973	0.09	0.27
Danazol	*y*=6.8×10^5^*x*+1.4×10^4^	0.1-100	0.9964	0.08	0.25
Testosterone propionate	*y*=2.8×10^4^*x*-4.3×10^3^	0.5-100	0.9983	0.25	0.74
Cortisone	*y*=2.4×10^5^*x*+1.1×10^4^	0.5-100	0.9961	0.15	0.46
Flumequine	*y*=2.4×10^4^*x*-5.6×10^2^	1.0-100	0.9983	0.27	0.81
Sparfloxacin	*y*=7.9×10^4^*x*-4.0×10^4^	0.5-200	0.9982	0.20	0.62
Atorvastatin calcium	*y*=2.2×10^5^*x*-4.6×10^5^	1.0-200	0.9970	0.15	0.46
Nitrendipine	*y*=1.4×10^4^*x*+1.1×10^4^	1.0-200	0.9998	0.28	0.84

*y*: quantitative ion peak area; *x*: mass concentration, μg/L.

### 2.7 回收率及精密度

采用标准添加法,以空白芽苗菜样品为添加对象,准确添加27种PPCPs标准物质,使得样品中化合物含量分别为4.0、40、160 μg/kg,涡旋混匀,静置30 min,按照1.3节和1.4节相关操作进行前处理以及定量分析。结果显示,芽苗菜的平均加标回收率为80.8%~122.3%,RSD为1.0%~9.9%(见表4),表明该方法具有良好的回收率及精密度。

**表4:** 27种PPCPs的回收率及精密度(*n*=6)

Drug	Spiked/ (μg/kg)	Recovery/%	RSD/%
Nisoldipine	4.0/40/160	93.6/101.3/96.1	5.1/3.9/5.6
Minoxidil	4.0/40/160	115.3/97.0/90.8	3.3/6.8/7.1
Nimodipine	4.0/40/160	86.0/84.5/90.7	4.8/3.6/5.4
Nicardipine	4.0/40/160	91.8/103.6/99.6	7.3/5.6/4.0
hydrochloride			
Indapamide	4.0/40/160	86.6/94.1/98.5	3.7/5.1/4.4
Lovastatin	4.0/40/160	95.7/92.1/91.1	1.8/1.0/5.4
Mevastatin	4.0/40/160	106.3/84.6/90.3	9.0/3.4/1.2
Amantadine	4.0/40/160	101.2/85.3/98.4	2.4/2.6/2.2
Diphenhydramine	4.0/40/160	101.0/90.8/88.6	9.7/8.7/9.9
Chlorpheniramine	4.0/40/160	94.8/90.8/105.1	9.6/4.4/3.2
maleate			
Carbamazepine	4.0/40/160	96.5/90.5/86.3	7.1/9.5/5.1
Sibutramine	4.0/40/160	120.4/94.9/110.1	3.0/4.5/7.7
Haemosibutramine	4.0/40/160	103.8/91.6/95.6	7.6/4.3/6.8
Chlorosibutramine	4.0/40/160	94.8/90.8/93.4	1.9/2.5/7.4
*N*-Monodesmethyl	4.0/40/160	122.3/103.7/96.7	8.1/3.0/4.2
sibutramine			
*N*,*N*-Bis-desmethyl	4.0/40/160	81.7/96.2/96.3	4.6/8.0/6.5
sibutramine			
Glibenclamide	4.0/40/160	97.6/93.5/102.3	7.8/3.3/3.1
Glimepiride	4.0/40/160	107.1/84.8/111.2	4.9/7.2/3.8
Gliclazide	4.0/40/160	97.7/100.6/89.6	5.7/6.6/8.3
Repaglinide	4.0/40/160	80.8/86.9/90.6	3.9/2.2/5.3
Danazol	4.0/40/160	93.6/106.2/93.8	2.4/4.2/3.2
Testosterone	4.0/40/160	110.0/113.8/108.3	4.6/3.1/1.6
propionate			
Cortisone	4.0/40/160	103.9/84.8/90.8	2.2/5.9/6.4
Flumequine	4.0/40/160	86.9/91.1/95.3	2.6/5.2/4.0
Sparfloxacin	4.0/40/160	86.4/84.5/88.6	3.9/2.7/5.4
Atorvastatin	4.0/40/160	95.3/88.6/94.6	3.0/5.8/1.8
calcium			
Nitrendipine	4.0/40/160	103.3/94.3/90.5	2.3/4.8/7.5

### 2.8 芽苗菜中PPCPs分析

#### 2.8.1 芽苗菜中PPCPs含量测定

在恒温恒湿植物培养箱中,采用0.5、5、50 μg/L 3个水平的PPCPs培养液培育芽苗菜,12天后收获,用去离子水去除表面药物残留,将其按根、茎、叶分离,制备成植物匀浆。然后,按照1.3节、1.4节方法分析测定(见表5)。结果发现,在PPCPs培养液中生长的芽苗菜各组织中,检测到了马来酸氯苯那敏、豪莫西布曲明、氯代西布曲明、金刚烷胺、苯海拉明、卡马西平、*N*-单去甲基西布曲明、西布曲明、*N*,*N*-双去甲基西布曲明、格列苯脲、瑞格列奈、氟甲喹、阿托伐他汀钙、盐酸尼卡地平、米诺地尔、尼莫地平、美伐他汀、格列美脲、司帕沙星,表明植物对PPCPs的吸收具有普遍性和广泛性,这与Carter等^[26]^和Knight等^[27]^在2018年所发表的研究结论相类似;而尼索地平、吲达帕胺、洛伐他汀、格列齐特、达那唑、丙酸睾酮、可的松、尼群地平在0.5、5、50 μg/L 3个水平的PPCPs培养液中生长的芽苗菜中均未检出,表明芽苗菜对PPCPs的吸收具有选择性和差异性,这一现象或许与植物的脂质和碳水化合物的含量、PPCPs自身理化性质以及培养环境有着密切联系^[10,28]^。从表5还可以看出,PPCPs在植物各器官中的含量有着明显的差异,除豪莫西布曲明(叶>茎>根)、格列苯脲(根>叶>茎)外,其余吸收的PPCPs在植物组织间的含量分布均为根>茎>叶,表明植物对PPCPs的吸收始于根部,并易于根部积累,一旦进入植物根部,PPCPs存在向植物茎、叶、果实积累的风险,或许与Mille等^[29]^所报道的植物通过被动扩散将溶解的PPCPs等有机物吸收,从而导致在植物根部有大量药物积累的情况相近。因此,如果蔬菜生长的环境以及灌溉用水受到PPCPs等污染物的污染,可能会在植物的各个组织器官中积累,对人类食品安全带来潜在危害。

**表5:** 在0.5、5、50 μg/L的PPCPs培养基中生长的芽苗菜组织(根、茎、叶)中PPCPs的含量

Drug	0.5 μg/L				5 μg/L				50 μg/L			
	root/ (μg/kg)	stem/ (μg/kg)	leaf/ (μg/kg)		root/ (μg/kg)	stem/ (μg/kg)	leaf/ (μg/kg)		root/ (μg/kg)	stem/ (μg/kg)	leaf/ (μg/kg)	
Chlorpheniramine maleate	1.8	1.2	0.2		15.4	14.2	0.8		143.8	83.8	10.2	
Haemosibutramine	0.2	0.4	1.3		1.5	2.2	2.6		21.3	23.1	26.6	
Chlorosibutramine	0.7	0.6	0.3		1.3	1.0	0.5		12.2	8.3	7.1	
Amantadine	1.2	0.9	-		8.2	4.3	-		84.3	34.6	1.4	
Diphenhydramine	1.0	0.6	-		6.9	2.9	0.2		69.6	22.1	1.5	
Carbamazepine	0.5	-	-		2.6	0.6	-		31.2	7.0	0.7	
*N*-Monodesmethyl sibutramine	0.4	0.3	-		4.6	2.2	-		53.5	16.8	1.0	
Sibutramine	-	-	-		3.5	2.1	0.5		37.5	18.3	4.0	
*N*,*N*-Bis-desmethyl sibutramine	-	-	-		1.9	0.6	-		29.2	5.0	-	
Glibenclamide	0.3	-	-		1.3	-	-		43.2	1.0	1.8	
Repaglinide	-	-	-		0.5	-	-		11.0	0.4	0.4	
Flumequine	4.5	-	-		10.7	-	-		226.8	7.1	3.6	
Atorvastatin calcium	0.9	-	-		4.9	-	-		98.3	1.4	1.3	
Aicardipine hydrochloride	-	-	-		-	-	-		6.4	0.4	5.1	
Minoxidil	-	-	-		-	-	-		2.4	-	-	
Nimodipine	-	-	-		-	-	-		3.1	-	-	
Mevastatin	-	-	-		-	-	-		3.0	-	-	
Glimepiride	-	-	-		-	-	-		7.3	-	-	
Sparfloxacin	-	-	-		-	-	-		5.1	-	-	
Nisoldipine	-	-	-		-	-	-		-	-	-	
Indapamide	-	-	-		-	-	-		-	-	-	
Lovastatin	-	-	-		-	-	-		-	-	-	
Gliclazide	-	-	-		-	-	-		-	-	-	
Danazol	-	-	-		-	-	-		-	-	-	
Testosterone propionate	-	-	-		-	-	-		-	-	-	
Cortisone	-	-	-		-	-	-		-	-	-	
Nitrendipine	-	-	-		-	-	-		-	-	-	

-: not detected.

#### 2.8.2 培养液中PPCPs含量对植物吸收的影响

通过芽苗菜根部的PPCPs含量分析发现,随着培养液中PPCPs含量升高,芽苗菜根部吸收药物的种类及含量也逐渐增加,且增长趋势十分明显(见图5)。表明植物根对药物的吸收量与环境中的污染物水平有着密切的关系,当环境中污染物含量达到一定水平,则会进入到植物体内,并在植物体内蓄积;这种趋势会随着外界PPCPs的含量上升而愈加明显。

**图5:**
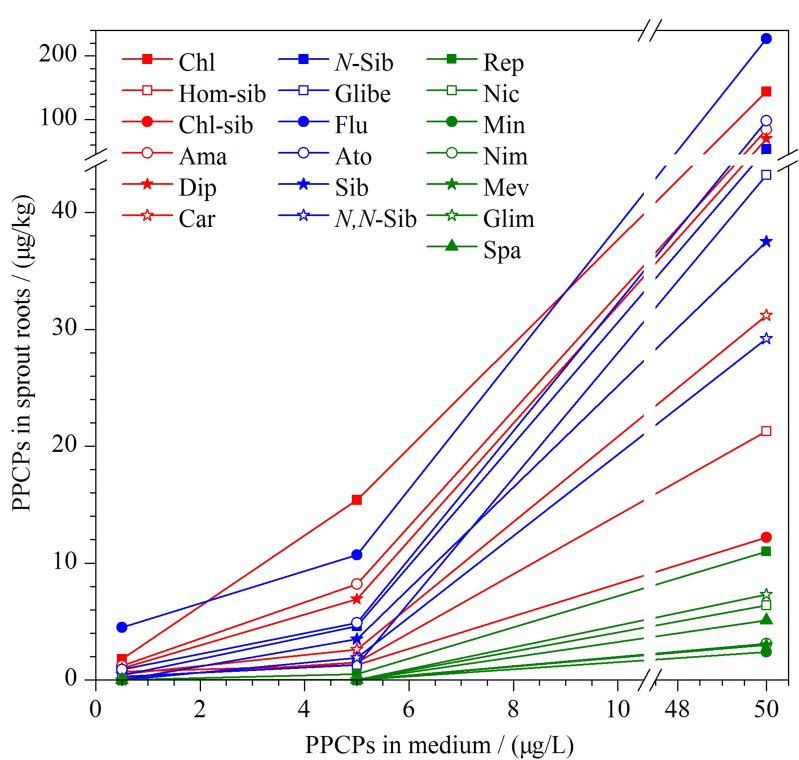
在0.5、5、50 μg/L 3个水平的PPCPs培养基 中生长的芽苗菜根中PPCPs的含量

#### 2.8.3 植物体内PPCPs的迁移

污染物被植物根系吸收后,植物会通过自身循环系统,将污染物转移至其他组织器官并积累,使得植物其他组织器官受到影响^[30]^。本研究分析了培养在50 μg/L PPCPs培养液中生长的芽苗菜所吸收的化合物,通过计算植物体内化合物的迁移因子(TF),评估植物体内化合物的转移情况,TF值越高,表明化合物在植物体内的迁移越活跃,植物吸收后很有可能迁移到植物根部以外的组织器官中。

TF=植物茎和(或)叶中化合物含量/植物根中化合物含量^[31]^。

豪莫西布曲明的TF=2.34,氯代西布曲明的TF=1.25,TF值均大于1,表明两种化合物在植物体内的迁移最为活跃;其次是盐酸尼卡地平、马来酸氯苯那敏、西布曲明、金刚烷胺、苯海拉明、*N*-单去甲基西布曲明、卡马西平等;而司帕沙星、米诺地尔、尼莫地平、美伐他汀、格列美脲的TF值为0(见图6),表明在50 μg/L PPCPs培养液中,PPCPs被根吸收后并未发生迁移,但并不能说明这些化合物在植物体内不发生迁移,或许与PPCPs自身理化性质、含量、药物接触周期、植物本身的脂质和碳水化合物差异以及外界环境温度、酸碱度有一定关系^[31⇓-33]^。

**图6:**
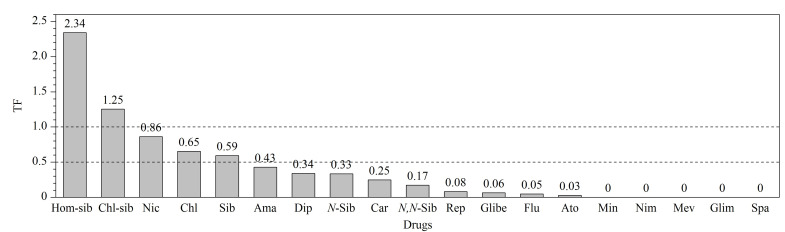
芽苗菜体内PPCPs在各组织中的迁移因子

## 3 结论

利用超高效液相色谱-串联质谱建立了测定植物体内27种PPCPs残留的分析方法。本方法前处理简单,有机溶剂消耗少,分析速度快,准确度及灵敏度较高,能满足植物中化合物的多残留分析以及环境监测。同时,以芽苗菜为研究对象,探讨了PPCPs在植物体内的吸收、迁移及积累情况。结果表明,植物对PPCPs的吸收积累量与外界PPCPs的含量有着显著联系,环境中污染物含量越高,植物体内污染物的含量及种类越多,且不同部位的富集量不同。因此,对环境中污染物及污染物水平加以严格监测,改进对生活污水的处理工艺,防止污水进入农牧业养殖以及人类生产生活十分关键。同时,PPCPs能够通过植物吸收进入到植物体内,对人类食品安全带来潜在的威胁,应对PPCPs新型污染物的发生与发展加以监测与管理。
